# An Account of Diseases in an Eastern District of London from December 20, 1809, to January 20, 1810

**Published:** 1810-02

**Authors:** 


### *An Account of Diseases in an Eastern District of London from December 20, 1809, to January 20, 1810.*

| | | | |
|---|---|---|---|
| ACUTE DISEASES. | | Menorrhagia | 4 |
| Typhus Mitior | 4 | Fluor Albus | 7 |
| Pneumonia | 7 | Diarrhœa | 3 |
| Pleuritis | 3 | Enterodynia | 4 |
| Peripneumonia Notha | 5 | Rheumatismus Chronicus | 12 |
| Rheumatismus Acutus | 3 | PUERPERAL DISEASES. | |
| CHRONIC DISEASES. | | Menorrhagia Lochialis | 3 |
| Tussis | 23 | Dysuria | 4 |
| Dyspnœa | 10 | Rhagas Papillæ | 5 |
| Tussis cum Dyspnœa | 28 | Dolores Post Partum | 7 |
| Hydrothorax | 3 | INFANTILE DISEASES. | |
| Anasarca | 2 | Aphthæ | 4 |
| Cephalalgia | 5 | Diarrhœa | 5 |
| Syncope | 1 | Dyspnœa | 2 |
| Hysteralgia | 1 | Convulsio | 3 |
| Gastrodynia | 4 | Vermes | 2 |

The changes in the temperature of the air, which have taken place within the last few weeks, have been very considerable, and in general very sudden. In the course of a few fours, the thermometer has shown a variation of 15 degrees. For a considerable time the wind blew from the west and south-west points, the temperature of the air was comparatively high, and a considerable degree of humidity prevailed.

Those diseases of the chest, which are the usual consesequence of the approach of winter, have assumed a milder form, and the disease wore more the appearance of the chronic than the acute species. During this period the cough and dyspnœa, or slight peripneumony of the aged, consiconsiderably abated. Amongst the instances included in the list, there was one of a man of 96 years of age, who recovered from a peripneumonia notha, and who is now as well as he has been for the last 20 years of his life.

During the state of the weather now referred to, fevers of the low or milder typhous kind prevailed. In one of the cases the disease was protracted to a very considerable length. The subject was a boy 10 years old, in whom the great prostration of strength and low delirium, attended with constant incoherent mutterings, strongly marked the nature of the disease. The weather having undergone so material a change within the last few days, we may expect an alteration in the state of diseases, and that in the complaints of the chest, the more acute and inflammatory species will prevail, attended with an aggravation of symptoms, which, with respect to the aged in particular, must produce considerable alarm.

